# Treatment of multidrug-resistant tuberculosis with bedaquiline, pretomanid, linezolid, and moxifloxacin following near-total gastrectomy

**DOI:** 10.1128/asmcr.00027-26

**Published:** 2026-08-05

**Authors:** Ihab Weheba, Sarah K. Brode, Melissa Richard-Greenblatt, Natasha F. Sabur

**Affiliations:** 1 Division of Respirology, University of Toronto 7938, Toronto, Ontario, Canada; 2 Division of Pulmonary Medicine, King Fahd Military Medical Complex 37841, Dhahran, Saudi Arabia; 3 University Health Network 7989, Toronto, Ontario, Canada; 4 Department of Pediatric Laboratory Medicine, The Hospital for Sick Children 7979, Toronto, Ontario, Canada; 5 Department of Laboratory Medicine and Pathobiology, University of Toronto 233837, Toronto, Ontario, Canada; 6 St. Michael’s Hospital, Unity Health Toronto 508783, Toronto, Ontario, Canada; Rush University Medical Center, Chicago, Illinois, USA

**Keywords:** gastrectomy, BPaLM, tuberculosis, multidrug-resistant

## Abstract

**Background:**

Patients with a history of gastrectomy are at increased risk of developing pulmonary tuberculosis due to nutritional deficiencies and potential immune impairment. Altered gastrointestinal anatomy can affect oral drug absorption and treatment pharmacokinetics, making management of drug-resistant tuberculosis in this population challenging.

**Case Summary:**

We describe a case of a woman with a history of gastric adenocarcinoma treated with chemotherapy, followed by near-total gastrectomy and Roux-en-Y reconstruction, who subsequently developed multidrug-resistant pulmonary tuberculosis. A biopsy of a pulmonary nodule was sent for culture and grew *Mycobacterium tuberculosis* complex. Phenotypic drug susceptibility testing could not be performed due to poor growth; however, a line probe assay performed on the isolate identified resistance to rifampin and isoniazid. The patient was treated with the all-oral bedaquiline, pretomanid, linezolid, and moxifloxacin (BPaLM) regimen. Due to concerns regarding impaired drug absorption following gastrectomy, therapeutic drug monitoring was performed to guide dosing. Therapeutic drug levels were ultimately achieved, and sputum culture conversion occurred after 5 weeks of effective therapy. The patient successfully completed the 26-week regimen with sustained microbiologic cure.

**Conclusion:**

This case demonstrates successful use of the all-oral BPaLM regimen in a patient with prior gastrectomy when supported by therapeutic drug monitoring. Evaluating drug exposure may help optimize treatment outcomes in patients at risk for drug malabsorption.

## INTRODUCTION

Malnutrition and prior gastric resection are known to be risk factors for the development of tuberculosis (TB) (1, 2). Malnutrition itself can increase susceptibility to infection by compromising the immune response to TB (3–5), and malabsorption of essential nutrients following gastrectomy, including vitamin B12 and iron, may impair host immunity (3, 6, 7). The incidence of tuberculosis among individuals who have undergone prior gastrectomy ranges from 0.4% to 5.0% (8). Gastrectomy patients are known to have a nearly twofold greater TB incidence compared to matched pre-operative controls (9).

In 2022, the World Health Organization (WHO) endorsed the use of a 6-month, all-oral BPaLM treatment regimen consisting of bedaquiline, pretomanid, linezolid, and moxifloxacin for treatment of multidrug-resistant pulmonary tuberculosis (MDR-TB) (10–13). However, the pharmacokinetic and pharmacodynamic properties of the newer agents remain incompletely characterized. In patients with prior gastrectomy, oral drug exposure may be altered through several mechanisms, including reduced gastric reservoir capacity, accelerated gastric emptying, reduced gastric acid secretion (hypochlorhydria), and changes in bile salt physiology following Roux-en-Y reconstruction (14–16). These physiological alterations may impair drug dissolution and intestinal absorption, resulting in reduced bioavailability of orally administered anti-TB medications (16–18).

Although the BPaLM agents are not thought to require gastric acid for activation, altered gastrointestinal physiology may influence their absorption and systemic drug exposure (16, 17). For this reason, intravenous treatments have traditionally been favored in this population, and the WHO has highlighted the importance of ensuring access to intravenous TB therapies for managing cases when oral absorption is compromised (19). However, as traditional MDR-TB regimens are longer and associated with significant adverse events, the ability to use the BPaLM regimen in this population would be beneficial.

Here, we describe a case of multidrug-resistant pulmonary tuberculosis in a patient with prior gastrectomy who was successfully treated with the all-oral BPaLM regimen. Therapeutic drug monitoring was helpful in ensuring that treatment was optimized and completed successfully.

## CASE PRESENTATION

A woman in her 30s, originally from North Africa, underwent routine CT screening in February 2023 for cancer surveillance. This revealed new pulmonary nodules with a tree-in-bud appearance, prompting concern for recurrent gastric cancer.

She had a history of gastric adenocarcinoma treated in 2017 with FLOT chemotherapy consisting of fluorouracil 2,600 mg/m² continuous infusion and leucovorin 200 mg/m², oxaliplatin 85 mg/m², and docetaxel 50 mg/m² given intravenously every 2 weeks, followed by near-total gastrectomy with Roux-en-Y gastrojejunostomy. She had been in remission subsequently but underwent continued regular medical surveillance. She had no prior history nor any known exposure to tuberculosis.

While awaiting workup, she developed intermittent fevers and began experiencing weight loss. A CT-guided biopsy of a pulmonary nodule demonstrated granulomatous inflammation, with the presence of acid-fast bacilli. A laboratory-developed multiplex *Mycobacterium tuberculosis* complex/*Mycobacterium avium* complex real-time polymerase chain reaction assay (20) was performed on the lung tissue, and deoxyribonucleic acid from *M. tuberculosis* complex was detected. Sputum samples were also collected and were 3+ acid-fast bacilli smear-positive. All subsequent cultures grew *M. tuberculosis* complex. Due to the absence of any known recent exposures and immune suppression following gastrectomy, it is likely that this case represented reactivated tuberculosis in our low-incidence setting.

She was initiated on standard first-line quadruple tuberculosis therapy, consisting of isoniazid 300 mg, rifampin 600 mg, pyrazinamide 1,000 mg, and ethambutol 800 mg, all taken once daily, based on a body weight of 43 kg. Despite growth of *M. tuberculosis* complex in both tissue biopsy and sputum samples, phenotypic drug susceptibility using the BACTEC Mycobacterium Growth Indicator Tube 960 system could not be completed due to poor growth. Alternative phenotypic drug susceptibility testing methods were unavailable, and therefore, the GenoType MTBDRplus version 2.0 assay (Hain Lifescience GmbH, Nehren, Germany) was performed on one of the sputum isolates 3 weeks into treatment. Resistance mutations to both rifampin and isoniazid were detected, suggesting multidrug-resistant pulmonary TB. She was referred to our tertiary tuberculosis treatment center for specialized MDR-TB management.

She was admitted to the inpatient tuberculosis unit, where she was started on a bridging regimen consisting of oral ethambutol 600 mg daily, moxifloxacin 400 mg daily, linezolid 600 mg once daily, and intravenous amikacin 450 mg daily. Health Canada approval for the use of bedaquiline and pretomanid was received after 2 weeks of bridging therapy. Amikacin and ethambutol were discontinued, and the patient was transitioned to the all-oral BPaLM regimen consisting of bedaquiline 400 mg daily (loading dose) followed by 200 mg thrice weekly, pretomanid 200 mg daily, linezolid 600 mg daily, and moxifloxacin 400 mg daily.

The patient continued to have intermittent fevers, and sputum culture conversion had not yet occurred despite 4 weeks of appropriate MDR-TB therapy (including 2 weeks of bridging therapy and 2 weeks of BPaLM). Given the history of prior gastrectomy, drug malabsorption of oral medications was considered. Therapeutic drug monitoring was therefore performed approximately 3 weeks after initiation of the BPaLM regimen, with all regimen components assessed simultaneously. Samples were analyzed at the University of Florida Infectious Diseases Pharmacokinetics Laboratory (Table 1). Moxifloxacin and bedaquiline levels were found to be appropriate. Pretomanid trough and peak levels were slightly higher than typical; after discussion with mycobacteriology experts at the University of Florida Infectious Diseases Pharmacokinetics Laboratory, the recommendation was made to continue at the current treatment dose while closely monitoring for adverse effects, particularly prolonged corrected QT interval. Linezolid peak levels were within the normal range (12–26 μg/mL), but trough values exceeded the <2 µg/mL suggested serum drug concentration for once-daily dosing. Linezolid dosing frequency was therefore adjusted from daily to thrice weekly (Monday, Wednesday, and Friday). Follow-up therapeutic drug monitoring for linezolid was performed approximately 2 weeks after dose adjustment and demonstrated levels within the desired therapeutic range. Sputum culture conversion was achieved after 5 weeks from the initiation of effective multidrug-resistant pulmonary tuberculosis therapy.

**TABLE 1:** Therapeutic drug monitoring of BPaLM regimen with reference ranges and post-dose adjustment results in a woman with near-total gastrectomy and multidrug-resistant tuberculosis

Drug	Sampling time^*a*^	Measured concn (μg/mL)	Reference range^*b*^ (μg/mL)	Interpretation	Clinical action
Bedaquiline (week 3 of BPaLM)	Trough	0.67	0.73–0.96	Within range	None
	2 h post-dose	0.85	2.8–3.3	Low	None
	6 h post-dose^*c*^	3.2	2.8–3.3	Within range	None
Moxifloxacin (week 3 of BPaLM)	Trough^*d*^	–^*e*^	–	–	–
	2 h post-dose	2.73	3.0–5.0	Within range	None
	6 h post-dose	3.31	3.0–5.0	Within range	None
Pretomanid (week 3 of BPaLM)	Trough	3.88	1.0–2.4	Elevated	Monitor
	2 h post-dose	3.56	1.4–2.6	Elevated	Monitor
	6 h post-dose	6.40	2.3–4.3	Elevated	Monitor
Linezolid (week 3 of BPaLM)	Trough	2.82	<2.0	Elevated	Dose reduced
	2 h post-dose	17.44	12.0–26.0	Within range	Dose reduced
	6 h post-dose	13.98	12.0–26.0	Within range	Dose reduced
Linezolid (week 5 of BPaLM)	Trough	Trace	<2.0	Within range	None
	2 h post-dose	17.69	12.0–26.0	Within range	None
	6 h post-dose	11.60	12.0–26.0	Slightly low	None

^*a*^ Blood sampling for therapeutic drug monitoring was performed based on recommended strategy for the described drugs as trough, 2 and 6 h post-oral dose.

^*b*^ Reference ranges were obtained from the University of Florida Infectious Disease Pharmacokinetics Laboratory (21).

^*c*^ Bedaquiline peak concentration occurs 4–6 h post oral dose.

^*d*^ Trough sampling is not recommended for moxifloxacin (21).

^*e*^ –, not available.

The patient developed new gastrointestinal symptoms, including nausea and abdominal discomfort, which occurred after taking her TB medications and which she had not experienced prior to initiating tuberculosis treatment. This was rectified by adjusting the timing of medication administration, with moxifloxacin being administered in the morning on an empty stomach to prevent inhibition of absorption by dietary divalent cations, bedaquiline administered at lunch, and pretomanid and linezolid administered during dinner. Anti-emetic medications were also utilized.

The patient had baseline anemia, with a hemoglobin level of 112 g/L (120–160 g/L) and a mean corpuscular volume of 80 fL (80–100 fL). She remained on maintenance vitamin B12 (cyanocobalamin) supplementation with 1,000 mcg monthly intramuscular injections. After initiating medications, there was a notable decline in both hemoglobin and mean corpuscular volume to 93 g/L and 76 fL, respectively. A workup for anemia was initiated to determine whether the anemia was secondary to linezolid, represented anemia of chronic disease from underlying tuberculosis, reflected iron deficiency from malabsorption in light of prior gastric resection, or was related to worsening of known underlying vitamin B12 deficiency. She was found to have profound iron deficiency, with a serum iron level of 5 µmol/L (11–27 µmol/L), normal total iron binding capacity of 56 µmol/L (45–72 µmol/L), and transferrin saturation of 9% (20–50%). She received two doses of intravenous iron. Hemoglobin improved to 99 g/L at the time of discharge and continued to improve throughout the treatment course, reaching 131 g/L by the end of treatment.

She developed peripheral neuropathy with tingling sensations in her hands and feet accompanied by neuropathic pain. This was thought to be a side effect of linezolid and eventually resolved once linezolid dosing was adjusted based on the results of therapeutic drug monitoring. She was monitored throughout treatment with ophthalmology evaluations and did not develop any signs of ocular toxicity. Likewise, cardiac monitoring with regular electrocardiograms consistently showed sinus rhythm and no signs of cardiac toxicity.

**Fig 1:**
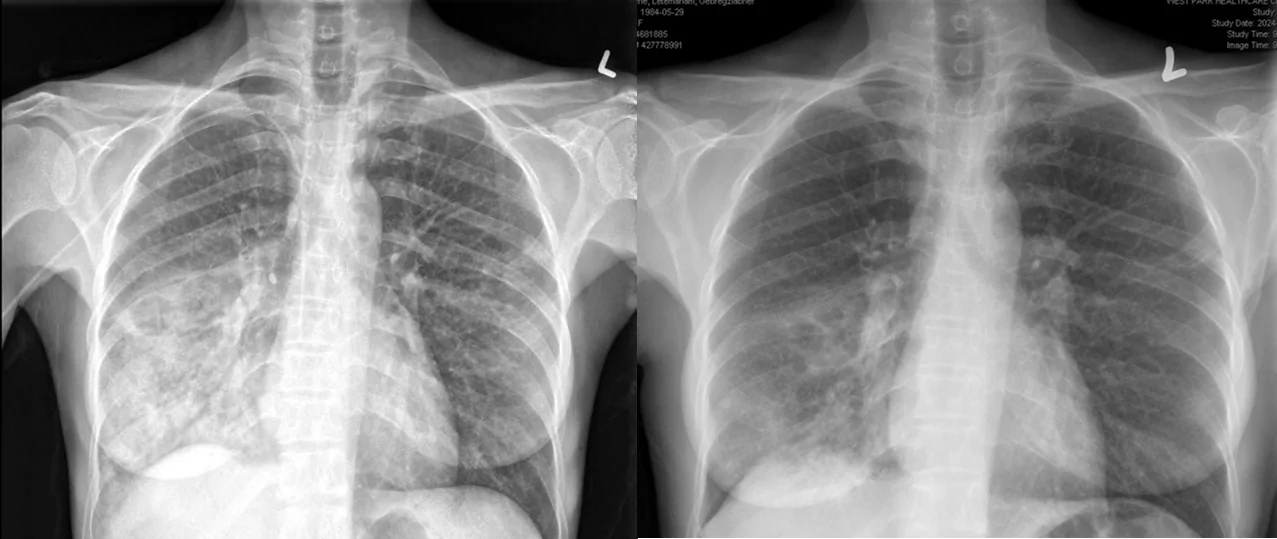
Serial chest radiographs demonstrating radiographic progression and residual disease following treatment of multidrug-resistant tuberculosis. (Left) Chest radiograph obtained at diagnosis (April 2025) demonstrating consolidation predominantly involving the right lower lobe with extension into the right middle lobe. (Right) Follow-up chest radiograph (February 2024) showing persistent right lower lobe airspace opacities with areas of lucency suggestive of evolving bronchiectasis.

**Fig 2:**
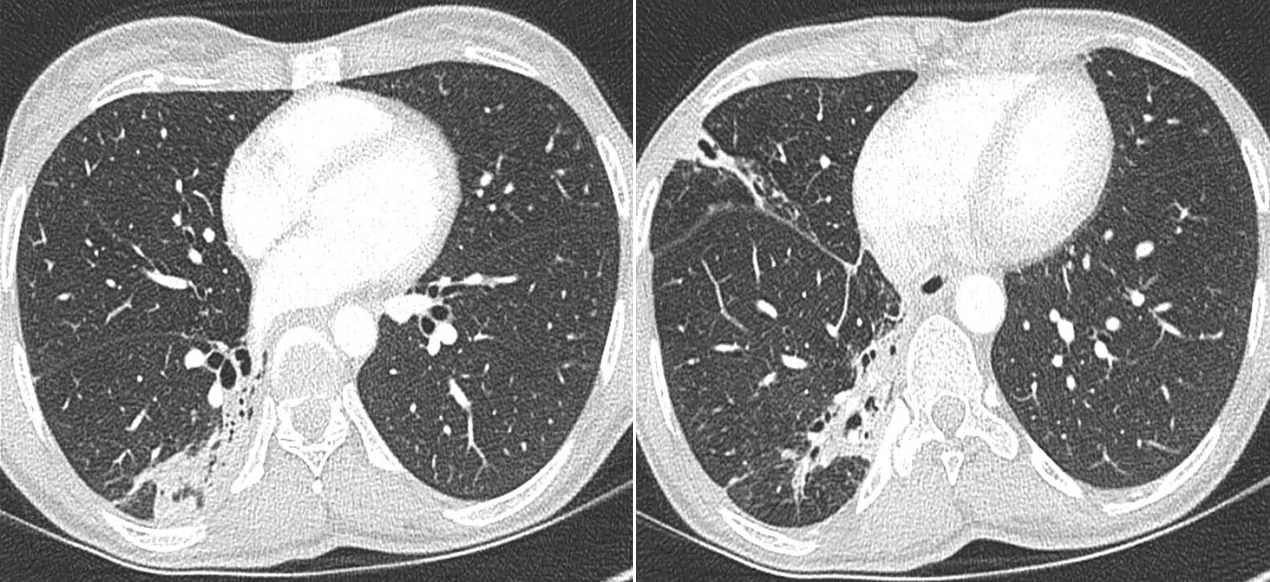
Computed tomography of the chest demonstrating post-treatment structural lung changes following multidrug-resistant tuberculosis. Patchy areas of consolidation, atelectasis, and bronchiectasis predominantly involving the right middle and lower lobes are consistent with sequelae of prior tuberculous disease.

Over the treatment course, there was progressive improvement in her symptoms, and she experienced a total weight gain of 3 kg. She successfully completed the 26-week BPaLM regimen in November 2023. Sputum mycobacterial cultures were negative at the end of treatment and remained negative at 18 months of follow-up. The end of treatment chest x-ray indicated definite improvement in the extensive right lower lobe airspace opacification; however, some airspace disease in the right lower lobe and areas of lucency suggestive of evolving bronchiectasis persisted (Fig. 1, left). A chest x-ray in February 2024 (Fig. 1, right) revealed persistent interstitial opacities and evolving bronchiectasis, confirmed on subsequent follow-up CT imaging (Fig. 2). Unfortunately, she developed recurrent gastric adenocarcinoma in early 2025 and passed away from complications related to recurrent cancer in the spring of 2025.

## DISCUSSION

Here we describe a case of multidrug-resistant pulmonary tuberculosis successfully treated with the BPaLM regimen in a patient with prior near-total gastrectomy. It is not well understood whether all-oral tuberculosis regimens can effectively be used in patients with a history of prior gastric resection. While some studies suggest that increasing the dosage of oral anti-TB drugs may improve outcomes in cases with malabsorptive disorders (22), the majority of available evidence supports the use of intravenous formulations in patients with documented malabsorption syndromes (23, 24).

A history of gastrectomy can impact both tuberculosis infection and treatment. Gastric cancer ranks as the fifth most prevalent cancer worldwide and remains primarily treated through surgery (25, 26). In a retrospective cohort study conducted on patients with gastric cancer undergoing gastrectomy, there was a significantly higher incidence of post-operative tuberculosis compared to the general population. Risk factors for post-gastrectomy TB included previous tuberculosis infection, lower body mass index, and extent of gastrectomy (1). Anemia following gastrectomy may result from iron deficiency and/or vitamin B12 deficiency, particularly in patients with altered post-operative gastrointestinal anatomy. These nutritional deficiencies may contribute to impaired immune function, increasing susceptibility to tuberculosis (14, 27). Subtotal or total gastrectomy may impact the absorption of anti-TB medications, and there are known to be changes in drug disposition after Roux-en-Y gastric bypass surgery (15, 16, 18, 28, 29).

The utilization of therapeutic drug monitoring (30–32) has gained widespread acceptance as a strategy to enhance the management of tuberculosis and is endorsed in the latest guidelines from the WHO and the American Thoracic Society, U.S. Centers for Disease Control and Prevention, European Respiratory Society, Infectious Diseases Society of America (ATS/CDC/ERS/IDSA) joint statement on drug-resistant TB treatment (12). Therapeutic drug monitoring in tuberculosis offers distinct advantages, including the prevention of toxicity, guidance in treating special patient populations, evaluation of therapy adherence, examination of potential drug interactions, and prevention of antimicrobial resistance (31, 33). In this case, therapeutic drug monitoring enabled the safe and effective use of the BPaLM regimen for multidrug-resistant pulmonary tuberculosis in the setting of prior gastrectomy. In our tertiary tuberculosis clinic, therapeutic drug monitoring is routinely performed for linezolid in all patients receiving the BPaLM regimen. In patients who are at risk for altered pharmacokinetics, including those with prior gastrointestinal resections or malabsorptive disorders, therapeutic drug monitoring for all BPaLM medications is performed after steady state is achieved (following the 2-week bedaquiline loading dose) and repeated following dose adjustments or if clinical concerns arise (12, 30, 31). In this patient, linezolid levels were assessed twice, once at baseline and then again after dose adjustment following elevated trough levels. Bedaquiline, pretomanid, and moxifloxacin levels were measured once after steady state was achieved, as no dose modification was required.

To our knowledge, this is the first described case of multidrug-resistant pulmonary tuberculosis successfully treated with the BPaLM regimen in a patient with prior near-total gastrectomy, suggesting that the BPaLM regimen can feasibly be used in patients with prior gastric resection. Our case also highlights the utility of using therapeutic drug monitoring to guide treatment in patients at risk for drug malabsorption.

### Conclusion

A history of gastrectomy may increase the risk of tuberculosis infection and reactivation. Subtotal or total gastrectomy may affect the absorption of anti-TB medications. In this patient with prior near-total gastrectomy, the short-course, all-oral BPaLM regimen was successfully used to treat multidrug-resistant pulmonary TB. Therapeutic drug monitoring was helpful to guide therapy and ensure adequate drug exposure. This case highlights the potential value of therapeutic drug monitoring in patients at risk for impaired drug absorption.
